# Editorial: Perioperative management and clinical challenges in elderly major surgical patients

**DOI:** 10.3389/fmed.2026.1987693

**Published:** 2026-09-21

**Authors:** Giuseppe Corazzelli, Sergio Corvino, Gennaro De Rosa, Francesco Fiore, Marco Filippelli, Luigi Sigona, Antonio Bocchetti, Salvatore Di Colandrea, Oreste de Divitiis, Carlo Gabriele Tocchetti, Alfonso Fiorelli, Mauro Mormile, Alessandra Cuomo, Plinio Cirillo, Sergio Paolini, Gualtiero Innocenzi, Vincenzo Esposito, Raffaele de Falco

**Affiliations:** 1 Department of Neurosurgery, Santa Maria Delle Grazie Hospital, ASL Napoli 2 Nord, Pozzuoli, Italy; 2 Department of Human Neurosciences, Sapienza University of Rome, Rome, Italy; 3 Department of Neurosurgery, Presidio Ospedaliero “Ospedale del Mare”, Naples, Italy; 4 Department of Advanced Biomedical Sciences, University of Naples Federico II, Naples, Italy; 5 Department of Translational Medical Sciences, University of Naples Federico II, Naples, Italy; 6 Department of Clinical Medicine and Surgery, University of Naples Federico II, Naples, Italy; 7 Department of Anaesthesiology and Intensive Care Medicine, Santa Maria Delle Grazie Hospital, ASL Napoli 2 Nord, Pozzuoli, Italy; 8 Department of Neurosciences and Reproductive and Odontostomatological Sciences, Neurosurgical Clinic, University of Naples Federico II, Naples, Italy; 9 Department of Translational Medicine, Thoracic Surgery Unit, University of Campania “Luigi Vanvitelli”, Naples, Italy; 10 Department of Neurosurgery, IRCCS Neuromed, Pozzilli, Italy

**Keywords:** editorial, frailty, geriatric surgery, multidisciplinary care, older surgical patients, perioperative care, postoperative complications

## 1 Introduction

Population aging is reshaping contemporary medical and surgical practice. Progressively, an increasing number of older adults undergo major surgical procedures ranging from orthopedic and abdominal surgery to complex oncological, cardiovascular, neurovascular, and spinal interventions. These patients frequently present with multimorbidity, frailty, reduced physiological reserve, cognitive vulnerability, and chronic pharmacological treatments, all of which may influence perioperative risk and recovery. However, the outcomes in older surgical patients not only depend on procedural success, but also on the appropriate patient selection, preoperative optimization, individualized anesthetic and hemodynamic management, prevention of postoperative complications, and effective functional rehabilitation (1).

In this setting, chronological age alone does not provide an omnicomprehensive representation of surgical risks (2, 3). Several measures of patient's frailty, nutritional and functional status, muscle strength, cognition, inflammation, and comorbidities may offer clinically meaningful estimates of biological vulnerability, guiding the surgical equipe towards the optimal reduction of the perioperative management (4).

At the same time, modifiable factors including anesthetic choice and dosage, pain control, hemodynamic stability, antithrombotic management, nutritional support, delirium prevention, nursing interventions, and timing of surgery create prospects to improve the functional and surgical outcomes throughout the perioperative pathway (5, 6). Furthermore, the older adult patients require a close collaboration between different professional clinical profiles, progressively configuring a multidisciplinary management to optimize every possible and thinkable risk probability (7, 8). Remarkably, the progressive development of updated clinical scoring and risk assessment measures suggest that the chronological age is progressively losing the indication not to proceed to major surgery.

Therefore, this Research Topic was conceived to configure a multidisciplinary framework for elderly patients, being the main focus of the research the moment of surgery, and the process throughout the pre-, peri-, and postoperative management of the increasing number of older adult major surgical patients.

## 2 Overview of the research topic

The Research Topic “Perioperative Management and Clinical Challenges in Elderly Major Surgical Patients” was developed to examine these challenges across surgical specialties and care settings. Its 43 contributions encompass original clinical studies, randomized and observational investigations, systematic and narrative reviews, meta-analyses, and case reports. Collectively, they provide a multidimensional account of contemporary geriatric surgery, extending from risk assessment and personalized perioperative management to complication prevention, functional recovery, and the appropriateness of advanced interventions in very old patients. Rather than defining advanced age as an absolute barrier to surgery, the Research Topic supports a risk-adapted, function-oriented, and patient-centered approach to perioperative decision-making.

To provide a coherent interpretation of this heterogeneous body of evidence, the contributions were discussed according to the successive phases of the perioperative pathway. The discussion therefore progressed from preoperative risk stratification and optimization to surgical indications, anesthetic and intraoperative management, postoperative complications, and functional recovery. Within each subsection, individual studies were integrated into a continuous thematic narrative rather than presented as isolated summaries. A final section addressed cross-cutting challenges in multidisciplinary and individualized decision-making, emphasizing how competing risks had to be balanced throughout the entire perioperative course. This structure reflected the continuity of care required by older surgical patients while highlighting the principal clinical themes that emerged from the Research Topic.

## 3 Discussion

### 3.1 Preoperative challenges

#### 3.1.1 Risk stratification and biological vulnerability

Risk stratification increasingly moved beyond chronological age toward direct measures of physiological reserve. In 195 patients undergoing intramedullary fixation of intertrochanteric fractures, Zhang et al. found that low preoperative handgrip strength was associated with delayed mobilization, longer hospitalization, more complications, poorer functional and quality-of-life outcomes, and higher 1-year mortality. Although its retrospective design precluded causal inference, the study supported handgrip strength as a simple, inexpensive marker capable of identifying patients who might benefit from intensified perioperative rehabilitation.

Biochemical indices offered a complementary assessment of vulnerability. In a retrospective cohort of 1,770 older patients undergoing hip fracture surgery, Jin et al. reported that a higher creatinine-to-albumin ratio independently predicted 1-year mortality [Hazard Ratio (HR) 1.49], integrating information on renal function, nutritional status, and systemic illness into an immediately available measure. This association remained exploratory but illustrated the potential value of routinely collected laboratory variables in preoperative prognostic assessment.

Frailty also encompassed frequently overlooked functional domains. Among 300 older adults scheduled for elective surgery, Wang et al. documented a 43% prevalence of oral frailty, which was associated with advanced age, female sex, general frailty, fewer natural teeth, and cognitive dysfunction. These findings placed oral function within comprehensive preoperative assessment, while the cross-sectional design did not establish its effect on postoperative outcomes.

The prognostic relevance of multidimensional frailty was further supported in pancreatic surgery. In a systematic review and meta-analysis of 11 studies, Li et al. found that frailty was associated with overall complications and a more than twofold increase in short-term mortality, whereas associations with major complications, readmission, and reoperation were not significant. Despite heterogeneity in frailty definitions and limited prospective evidence, these results indicated that biological vulnerability, rather than age alone, should inform patient selection and perioperative counseling before high-risk pancreatic procedures.

Population-level context remained equally important. In a six-year Ecuadorian series of 216 older adults, Miranda et al. showed that intertrochanteric fractures predominantly affected women and the oldest-old after low-energy falls, with frequent unstable patterns and a median preoperative stay of 5 days. The study highlighted both preventive priorities and the need to streamline access to surgery, although its single-center descriptive design limited broader inference.

Finally, Pan et al. systematically appraised 21 postoperative infection-prediction models derived from 17 hip-fracture studies. Despite apparently good discrimination, all included studies had a high risk of bias and poor adherence to prediction-model reporting standards, with limited external validation. Their critical synthesis therefore cautioned against premature clinical adoption and established multicenter prospective validation, transparent reporting, and rigorous calibration as prerequisites for reliable, individualized risk prediction.

#### 3.1.2 Preoperative optimization and surgical planning

Once vulnerability had been identified, preoperative assessment needed to be translated into targeted optimization without exposing older patients to unnecessary surgical delay. Using data from 15,429 adults admitted to intensive care after elective non-cardiac surgery, Berikashvili et al. compared 563 propensity-matched pairs with and without chronic obstructive pulmonary disease. Chronic Obstructive Pulmonary Disease (COPD) was not associated with higher hospital or intensive care mortality, but was linked to more frequent mechanical ventilation and a modestly longer hospital stay. Despite the retrospective use of older database records and the inclusion of a broader adult population, these findings supported anticipatory respiratory optimization and resource planning rather than exclusion from surgery based on COPD alone.

In 112 older adults undergoing hip fracture surgery, Liu et al. found moderate discrimination of the Caprini score model for postoperative deep-vein thrombosis [Area Under the Curve (AUC) 0.724]. In the randomized phase, risk-guided nursing combined with nutritional support reduced thrombosis from 50.0% to 17.9% and improved nutritional and functional outcomes. However, the small single-center sample and bundled intervention prevented attribution of benefit to either component.

Protocolized care also addressed respiratory complications. In a before-and-after study involving 104 older patients with hip fractures, Zhang et al. translated synthesized evidence into a multidisciplinary hypoxemia prevention and management pathway. Implementation improved postoperative oxygen saturation, reduced hypoxemia on the first two postoperative days, and increased healthcare professionals' knowledge. The absence of randomization, short monitoring period, and uniform use of regional anesthesia limited generalizability, but the findings demonstrated the feasibility of converting evidence into standardized bedside practice.

Among 168 patients undergoing arthroplasty for displaced femoral neck fractures, He et al. associated surgery within 48 h with fewer 30-day complications, shorter hospitalization, and faster multidimensional recovery, without differences in readmission or 1-year mortality. Residual confounding by medical stability limited causal interpretation, supporting timely surgery after appropriate optimization rather than indiscriminate acceleration.

### 3.2 Surgical and intraoperative challenges

#### 3.2.1 Surgical indications and procedural appropriateness in advanced Age

Chronological age alone provided an insufficient basis for determining procedural appropriateness, particularly following the expansion of minimally invasive and catheter-based interventions. In a single-arm meta-analysis of 29 studies including 2,773 patients with pure severe native aortic regurgitation, Zhu et al. reported a device-success rate of 87.5%, with perioperative all-cause and cardiovascular mortality rates of 3.1% and 1.4%, respectively. Outcomes varied according to surgical risk, valve design, procedural approach, and geographical setting. Although the absence of a comparator and the observational nature of the available evidence precluded definitive estimates of comparative effectiveness, the findings supported transcatheter aortic valve replacement (TAVR) as a therapeutic option for carefully selected patients at high surgical risk.

The relevance of age-based selection was also examined in electrophysiology. In a real-world cohort of 217 consecutive patients undergoing contemporary atrial fibrillation ablation, De Rosa et al. found no independent association between age, analyzed both continuously and using predefined thresholds, and procedural complications or arrhythmic outcomes within 48 h. Although the single-center design and ultra-early observation period did not permit assessment of long-term effectiveness, the study indicated that chronological age should not constitute an isolated criterion for excluding appropriately selected patients from ablation.

In a propensity-matched retrospective study of 87 patients aged at least 75 years, Li et al. found comparable short-term and 90-day outcomes after bridging and direct mechanical thrombectomy. Time to arterial puncture, neurological status at 24 h, and C-reactive protein at 7 days predicted poor functional outcome. Although the derived model showed high apparent discrimination, the small single-center sample and absence of external validation limited clinical applicability.

In a rare and highly complex presentation, Dong et al. described a 75-year-old woman who developed two episodes of acute ischemic stroke following inferior myocardial infarction and recovered after two emergency thrombectomies. Although no generalizable conclusions could be derived from a single case, the report illustrated the potential role of repeated endovascular rescue treatment within individualized multidisciplinary decision-making.

Evidence from elective urological surgery further supported procedure-specific selection. In a systematic review and meta-analysis of 15 observational studies encompassing 2,231 patients with large-volume benign prostatic hyperplasia, Hao et al. found that laser enucleation was associated with shorter operative and catheterization times, whereas robot-assisted simple prostatectomy was associated with a lower incidence of urinary incontinence and modestly greater long-term improvement in symptoms. Major complication rates were comparable. Nevertheless, heterogeneity and the observational design of the included studies prevented the identification of a universally preferable technique, supporting individualized selection according to anatomy, functional priorities, procedural expertise, and perioperative risk.

In a comparative analysis of patients undergoing holmium laser enucleation of the prostate (HoLEP) for symptomatic benign prostatic hyperplasia, Bakbak et al. reported durable symptomatic and functional improvement after holmium laser enucleation in 30 men aged at least 85 years. The oldest-old required longer catheterization and experienced more minor complications than patients aged 55 years or younger, but operative duration, hemoglobin reduction, and hospitalization were comparable. Despite the small sample and single-surgeon design, the findings suggested that advanced age alone did not preclude clinically meaningful benefit from HoLEP.

In 164 patients older than 70 years undergoing laparoscopic common bile duct exploration, Li et al. associated primary duct closure with shorter surgery and hospitalization, faster gastrointestinal recovery, lower costs, and fewer electrolyte disturbances and bile leaks than T-tube drainage. Although retrospective treatment selection precluded causal inference, the findings supported primary closure in older patients meeting appropriate anatomical and technical criteria.

#### 3.2.2 Anesthetic, hemodynamic, and intraoperative management

Age-related variation in anesthetic sensitivity and cardiovascular reserve underscored the need for individualized pharmacological and hemodynamic management. In a prospective cohort of 102 patients, Lu et al. found that participants aged at least 65 years required a lower ciprofol dose to achieve loss of consciousness than those aged 18–64 years, with age remaining independently associated with dose requirement after multivariable adjustment. Because the study included only ASA I–II patients, employed a single infusion regimen, and assessed induction rather than subsequent anesthetic requirements or clinical outcomes, its findings provided preliminary pharmacodynamic evidence for cautious age-adjusted titration rather than a definitive dosing recommendation.

In a prospective randomized trial of 80 patients aged 65–75 years undergoing orthopedic surgery, Dong et al. found no significant differences between desflurane and sevoflurane in Mini-Mental State Examination (MMSE) scores through 48 h, hemodynamic variables, adverse events, or opioid consumption. Desflurane was associated with shorter times to eye-opening, extubation, command following, and post-anesthesia care unit discharge. These findings supported an early recovery advantage, but did not establish superior cognitive protection, particularly because MMSE may have lacked sensitivity for subtle perioperative neurocognitive changes and later impairment was not assessed.

Sedation during positioning for spinal anesthesia was examined by Guo et al. in 94 older adults undergoing lower-extremity fracture surgery. S-ketamine combined with dexmedetomidine was associated with modestly lower pain and better posture quality during the most painful positioning stages than S-ketamine combined with propofol. However, clinician-directed treatment allocation, retrospective outcome ascertainment, use of an institution-specific posture scale, and limited power for adverse events restricted causal and safety inferences.

In an open-label randomized study of 60 older patients undergoing laparoscopic colorectal cancer surgery, Wang et al. found that remimazolam reduced vasopressor use but prolonged emergence. It did not improve the primary endpoint of natural killer-cell percentage at 24 h, while immediate post-extubation changes in T-cell subsets were transient. The small, unregistered study therefore provided no evidence of superior immune preservation or longer-term oncological benefit.

Proactive vasopressor administration was assessed by Zhao et al. in a retrospective cohort of 119 patients aged at least 80 years undergoing total hip arthroplasty. Continuous low-dose norepinephrine was associated with a lower burden of mean arterial pressure below 65 mmHg than conventional reactive management. Lower delirium incidence and shorter postoperative hospitalization were also observed, but these secondary associations remained susceptible to treatment-selection bias and unmeasured differences in anesthetic depth, fluid administration, temperature, and baseline cognitive status. The study therefore supported prospective evaluation of prophylactic norepinephrine rather than establishing its superiority.

In a retrospective pre-post pilot study of 48 patients undergoing pancreatic surgery, Piccioni et al. associated implementation of a Hypotension Prediction Index-guided protocol with higher mean arterial pressure and lower hypotension burden, without differences in postoperative outcomes. Concurrent protocol modifications, temporal confounding, and the small sample precluded attribution to the algorithm and limited the findings to feasibility and hypothesis generation.

Finally, Meng et al. synthesized eight randomized trials comprising 735 patients undergoing intramedullary fixation of intertrochanteric fractures. Intravenous tranexamic acid reduced hidden and total blood loss and transfusion requirements, with no statistically detectable increase in thromboembolic events. The limited cumulative sample, heterogeneous dosing and timing, and short follow-up in several trials prevented definitive assessment of uncommon or delayed adverse events. Nevertheless, among the contributions considered in this section, this meta-analysis provided the most consistent evidence for an effective intraoperative blood-conservation strategy. Overall, the available studies favored individualized anesthetic titration and proactive management of hemodynamic and hemorrhagic risk, while the clinical benefits of specific agents and predictive technologies remained to be confirmed in adequately powered trials.

### 3.3 Postoperative challenges and recovery

#### 3.3.1 Neurocognitive outcomes, sleep, and pain

In a retrospective cohort of 505 patients undergoing total knee arthroplasty, Zhang et al. compared ten machine-learning models for postoperative sleep disturbance. Gradient boosting showed the highest apparent discrimination (AUC 0.906). However, inclusion of pain and anxiety measured one month postoperatively introduced information unavailable at baseline; absent calibration and external validation further precluded interpretation as a preoperative prediction model. The findings therefore remained exploratory.

A perioperative nomogram was developed by Liu et al. from 248 patients undergoing internal fixation of intertrochanteric fractures after standardized preoperative dexamethasone administration. Age, preoperative monocyte count, and postoperative intensive care transfer predicted delirium, with an internally validated AUC of 0.803. Because the model included a postoperative variable, it supported dynamic perioperative risk reassessment rather than exclusively preoperative stratification. Its single-center retrospective derivation, 31 delirium events, wide confidence interval for monocyte count, record-based delirium ascertainment, and absence of external validation limited clinical deployment; moreover, the uniform dexamethasone exposure did not permit evaluation of its preventive efficacy.

Biological correlates of postoperative cognitive dysfunction were examined by Fu et al. in a prospective cohort of 361 patients undergoing cholecystectomy, from which 24 patients with cognitive decline were matched to 48 controls. Postoperative lipopolysaccharide-binding protein and inflammatory markers, particularly interleukin-6, were independently associated with cognitive dysfunction. These findings supported intestinal barrier injury and systemic inflammation as candidate components of the gut-brain pathway; however, the small number of cases, postoperative biomarker measurement, peripheral rather than central inflammatory assessment, and observational design neither established causal mediation nor validated a clinically applicable predictive strategy.

Anesthetic selection was evaluated by Ren et al. in a randomized trial of 90 older patients undergoing laparoscopic-assisted gastrointestinal surgery. Remimazolam, sevoflurane, and their combination produced no significant differences in delirium or delayed early cognitive recovery, whereas remimazolam was associated with less intraoperative hypotension and lower vasopressor requirements. With only 30 participants per group, restrictive eligibility criteria, and cognitive follow-up limited to three postoperative days, the trial could not exclude smaller but clinically relevant neurocognitive differences; its principal signal therefore concerned hemodynamic stability rather than demonstrated neuroprotection.

He et al. reported a lower incidence of delirium with fentanyl than with sufentanil among 178 older patients who developed perioperative ischemic stroke after non-cardiac surgery, with the association persisting after multivariable adjustment and propensity matching. However, retrospective treatment allocation and incomplete control of stroke, procedure-related, anesthetic, and temporal confounders precluded causal attribution. The findings therefore supported hypothesis generation rather than a definitive disadvantage of sufentanil.

The most extensive interventional evidence was synthesized by Wu et al. in a systematic review and meta-analysis of 26 randomized trials including 2,309 older patients. Perioperative electroacupuncture was associated with a lower incidence of perioperative neurocognitive disorders (Relative Risk 0.47), whereas improvements in cognitive scores and inflammatory or neuronal-injury biomarkers were accompanied by considerable heterogeneity. All trials were conducted in China, 80.8% had a high risk of performance bias because of inadequate blinding, outcome definitions and intervention protocols varied, and evidence certainty ranged from moderate to very low. Electroacupuncture therefore represented a promising preventive intervention, but its effect required confirmation through well-blinded, multicenter trials using standardized neurocognitive definitions and longer follow-up.

Nair et al. reviewed the potential roles of central sensitization, neuroinflammation, and psychosocial vulnerability in chronic postsurgical pain and prolonged opioid use, emphasizing preoperative phenotyping, multimodal opioid-sparing analgesia, and transitional pain services. However, the narrative methodology precluded quantitative assessment of comparative effectiveness, while several proposed epigenetic and microbiome mechanisms remained predominantly preclinical. The review nevertheless broadened perioperative care toward prevention of pain chronification and persistent opioid exposure.

#### 3.3.2 Postoperative complications and early clinical management

In a retrospective single-center cohort of 698 older patients with hip fracture and preoperative stroke, Li et al. developed a perioperative pneumonia model integrating eight clinical and laboratory variables. Discrimination declined from the training to the randomly partitioned validation cohort (AUC 0.920 vs. 0.736), suggesting substantial optimism. The omission of stroke severity, swallowing function, previous aspiration pneumonia, cognition, and opioid exposure further limited risk adjustment. Thus, despite internal evaluation, the model required prospective external validation before clinical implementation.

A simpler inflammatory-nutritional marker was examined by Shi et al. in 1,249 patients with hip fractures, 122 of whom developed postoperative pneumonia. A higher neutrophil percentage-to-albumin ratio (NPAR) was independently associated with pneumonia after multivariable adjustment and showed a linear dose-response relationship. However, its discrimination was modest (AUC 0.66), and relevant confounders, including pulmonary function, smoking, COPD, functional status, ASA classification, ventilatory support, and oral hygiene, were unavailable. NPAR could consequently be considered an accessible screening marker of combined inflammatory and nutritional vulnerability, but not a sufficiently accurate stand-alone predictor of individual pneumonia risk.

Nutritional complications were examined by Chen et al. in 346 older patients undergoing laparoscopic gastric cancer surgery. Early enteral nutrition intolerance was reported in 56.1% and was independently associated with age ≥75 years, advanced tumor stage, diabetes, radical resection, and intraoperative blood loss ≥250 mL. A five-factor score showed good apparent discrimination (AUC 0.856). However, the symptom-based definition may have inflated clinically relevant intolerance, as feeding required modification or discontinuation in only 29.9% of affected patients. The retrospective derivation-only design, without calibration or internal or external validation, precluded prescriptive use of the proposed nursing strategies.

#### 3.3.3 Functional recovery, rehabilitation, and transitional care

Functional recovery emerged as a multidimensional outcome shaped by physical reserve, symptom control, adherence to rehabilitation, and preparedness for the transition from hospital to home.

In a retrospective cohort of 325 older adults undergoing surgery for hip fracture, Yin et al. found that 81.2% of patients without pre-fracture sarcopenia risk had a Strength, Assistance with walking, rising from a chair, Climbing stairs, and Falls (SARC-F) score ≥4 three months after discharge. Older age, lower educational attainment, poorer activities of daily living, and impaired masticatory function were independently associated with a positive postoperative screen. Importantly, because sarcopenia was assessed exclusively through SARC-F rather than objective measures of muscle strength, mass, or physical performance, the findings quantified postoperative sarcopenia risk rather than its clinically confirmed incidence.

The relationship between postoperative pain management and functional recovery was examined by Xie et al. in a systematic review and meta-analysis of five studies comprising 539 patients after hip arthroplasty. Pain-relief-oriented nursing interventions were associated with lower pain intensity and better activities of daily living than conventional care. However, the small evidence base, combination of randomized and observational designs, and clinical heterogeneity of the multicomponent interventions limited both the certainty of the pooled estimates and the attribution of benefit specifically to pain-relief nursing.

In a randomized controlled trial including 80 older patients undergoing surgery for intertrochanteric femoral fracture, Zhang et al. compared routine care with an evidence-based nursing intervention informed by Watson's theory. The intervention was associated with greater rehabilitation adherence, lower postoperative pain, earlier weight bearing and fracture healing, better hip function and quality of life, and fewer complications. Although randomization strengthened the internal validity of the comparison, the small single-center sample, short follow-up, and composite nature of the intervention precluded determination of which component accounted for the observed effects and constrained external generalizability.

Liao et al. identified three discharge-readiness profiles in a cross-sectional study of 356 older inpatients with osteoporotic vertebral compression fractures. Profile membership was associated with comorbidity burden, length of stay, first hospitalization, and discharge-teaching quality, supporting differentiated transitional-care planning. However, the single-center design, inclusion irrespective of surgical treatment, and absence of longitudinal validation limited causal and prognostic interpretation.

### 3.4 Integrated perioperative decision-making

#### 3.4.1 Multidisciplinary and individualized management of complex risk

Complex perioperative decisions required integration of procedural indications with cognitive status, physiological reserve, functional objectives, and competing clinical risks.

Rao et al. reviewed the management of hip fracture in older adults with cognitive impairment, supporting timely surgery, orthogeriatric co-management, effective analgesia, delirium prevention, early mobilization, and cognitively adapted rehabilitation. By emphasizing heterogeneity in cognitive severity, frailty, and pre-fracture function, the review argued against therapeutic nihilism and favored individualized recovery goals. Its narrative design and the absence of formal risk-of-bias assessment or certainty grading, however, limited the strength of its recommendations.

In a retrospective cohort of 141 patients with failed internal fixation of peri-trochanteric fractures, Zhang et al. classified failure patterns according to mechanical instability, deformity, infection, implant integrity, and articular damage, linking each category to a differentiated revision strategy. Functional outcomes improved after revision, while arthroplasty was preferentially used in older patients or when articular preservation was no longer feasible. Although clinically informative, the proposed framework remained retrospectively derived and lacked independent validation; selection bias, unmeasured confounding, and small subgroups also precluded comparative conclusions regarding the superiority of individual strategies.

Jia et al. described a patient with thoracolumbar infection and cauda equina syndrome complicated by an extensive sacrococcygeal pressure injury. The case highlighted how neurological impairment, immobility, and sphincter dysfunction could obscure early clinical recognition and supported coordinated management of spinal infection and pressure-related tissue damage, without permitting conclusions on the comparative effectiveness of the adopted strategy.

Similarly, Chen et al. reported margin-negative pancreaticoduodenectomy in a carefully selected 90-year-old patient with distal cholangiocarcinoma and a tumor-associated granulomatous reaction. The report illustrated that chronological age alone did not determine surgical eligibility when physiological reserve, resectability, expected benefit, and patient preference were jointly considered, although a single favorable outcome could not establish general safety or long-term oncological effectiveness.

Finally, Corazzelli et al. evaluated perioperative antithrombotic management in a multicenter retrospective cohort of 767 consecutive patients aged ≥60 years undergoing elective degenerative spine surgery. Substitution with low-molecular-weight heparin or low-dose aspirin was not associated with clinically relevant differences in hemoglobin reduction, operative duration, hospitalization, or recorded complications. No clinically detected major hemorrhagic or thromboembolic events occurred. However, confounding by indication, pharmacological heterogeneity, absence of systematic screening for asymptomatic events, and limited power for rare complications precluded causal or definitive safety conclusions. The findings supported protocol feasibility in selected patients rather than equivalence between strategies.

## 4 Conclusion

The contributions to this Research Topic portrayed perioperative care in older surgical patients as a continuous and inherently multidisciplinary process. Across diverse surgical settings, chronological age alone did not adequately define operative risk, treatment eligibility, or expected recovery. Frailty, cognitive and functional status, nutritional reserve, comorbidity burden, procedural complexity, and patient-centered goals emerged as more informative determinants of clinical decision-making. The collected evidence also indicated that outcomes depended not only on technical success, but on coordinated risk assessment, physiological optimization, appropriate intraoperative management, prevention of postoperative complications, and timely rehabilitation. Nevertheless, the predominance of retrospective and single-center studies, heterogeneous outcome definitions, and limited external validation constrained the generalizability of several findings. Future research should therefore prioritize adequately powered prospective studies, standardized geriatric and perioperative outcomes, and validated decision-support tools. Such efforts will be essential to translate individualized multidisciplinary care into reproducible pathways capable of improving survival, functional independence, and quality of life in an increasingly complex surgical population.
